# CD20 as a gatekeeper of the resting state of human B cells

**DOI:** 10.1073/pnas.2021342118

**Published:** 2021-02-09

**Authors:** Kathrin Kläsener, Julia Jellusova, Geoffroy Andrieux, Ulrich Salzer, Chiara Böhler, Sebastian N. Steiner, Jonas B. Albinus, Marco Cavallari, Beatrix Süß, Reinhard E. Voll, Melanie Boerries, Bernd Wollscheid, Michael Reth

**Affiliations:** ^a^Biology III, Faculty of Biology, University of Freiburg, 79104 Freiburg, Germany;; ^b^Centre for Biological Signalling Studies, University of Freiburg, 79110 Freiburg, Germany;; ^c^Centre for Integrative Biological Signalling Studies, University of Freiburg, 79104 Freiburg, Germany;; ^d^Institut für Klinische Chemie und Pathobiochemie, Klinikum rechts der Isar, Technical University of Munich, 81675 Munich, Germany;; ^e^lnstitute of Medical Bioinformatics and Systems Medicine, Medical Center–University of Freiburg, Faculty of Medicine, University of Freiburg, 79110 Freiburg, Germany;; ^f^German Cancer Consortium (Deutsches Konsortium für Translationale Krebsforschung) Partner Site Freiburg, German Cancer Research Center (Deutsches Krebsforschungszentrum), 69120 Heidelberg, Germany;; ^g^Department of Rheumatology and Clinical Immunology, Medical Center–University of Freiburg, Faculty of Medicine, University of Freiburg, 79106 Freiburg, Germany;; ^h^Department of Health Sciences and Technology, Institute of Translational Medicine, Swiss Federal Institute of Technology, ETH Zürich, 8093 Zurich, Switzerland;; ^i^Wollscheid-Group, Swiss Institute of Bioinformatics, 1015 Lausanne, Switzerland;; ^j^Department of Biology, Centre for Synthetic Biology, Technical University Darmstadt, 64289 Darmstadt, Germany;; ^k^Comprehensive Cancer Center Freiburg, Medical Center–University of Freiburg, University of Freiburg, 79106 Freiburg, Germany

**Keywords:** B lymphocyte, therapeutic antibody, CD20, plasma cell

## Abstract

CD20 is a B cell-specific membrane protein and represents an attractive target for therapeutic antibodies. Despite widespread usage of anti-CD20 antibodies for B cell depletion therapies, the biological function of their target remains unclear. Here, we demonstrate that CD20 controls the nanoscale organization of receptors on the surface of resting B lymphocytes. CRISPR/Cas9-mediated ablation of CD20 in resting B cells resulted in relocalization and interaction of the IgM-class B cell antigen receptor with the coreceptor CD19. This receptor rearrangement led to a transient activation of B cells, accompanied by the internalization of many B cell surface marker proteins. Reexpression of CD20 restored the expression of the B cell surface proteins and the resting state of Ramos B cells. Similarly, treatment of Ramos or naive human B cells with the anti-CD20 antibody rituximab induced nanoscale receptor rearrangements and transient B cell activation in vitro and in vivo. A departure from the resting B cell state followed by the loss of B cell identity of CD20-deficient Ramos B cells was accompanied by a PAX5 to BLIMP-1 transcriptional switch, metabolic reprogramming toward oxidative phosphorylation, and a shift toward plasma cell development. Thus, anti-CD20 engagement or the loss of CD20 disrupts membrane organization, profoundly altering the fate of human B cells.

## Significance

Worldwide about one million patients are given anti-CD20 antibodies such as rituximab (RTX) for the treatment of B cell-associated diseases. Despite the success of this first therapeutic antibody, little is known about the function of its target. The role of CD20 only becomes clear in the context of the nanoscale compartmentalization of the B lymphocyte membrane. We found that CD20 is an organizer of the IgD-class nanocluster on the B cell membrane. The loss of CD20 on human B cells results in a dissolution of the IgD-class nanocluster and a transient B cell activation inducing a B cell-to-PC differentiation. Thus, CD20 is an essential gatekeeper of a membrane nanodomain and the resting state of naive B cells.

CD20 is an atypical tetraspanin expressed only on mature B cells. Due to its B cell-specific expression, CD20 is an ideal target of B cell-depleting monoclonal antibodies, most prominently rituximab (RTX). These anti-CD20 antibodies are frequently used in the clinic for the treatment of B cell tumors such as Burkitt lymphoma, non-Hodgkin lymphoma, and chronic lymphocytic leukemia (CLL) or B cell-associated autoimmune diseases like rheumatoid arthritis (RA) (1–4). RTX has also successfully been used to treat multiple sclerosis or systemic lupus erythematosus (5). Despite the undeniable therapeutic value of this treatment, the specific molecular events leading to successful B cell depletion after RTX administration are not fully understood (6). Although antibody-dependent cellular cytotoxicity (ADCC) or complement-dependent cytotoxicity seem to play a role in removing RTX-bound B cells in vivo, additional mechanisms of B cell elimination cannot be excluded (7, 8). Since many patients relapse after RTX treatment, there is a need for a deeper understanding of the role CD20 plays in B cell signaling and cell fate determination (6).

Mature B cells coexpress an IgM–class B cell antigen receptor (BCR) and an IgD-class BCR. Using several superresolution techniques, we previously showed that the two different BCR classes are localized on the surface of resting B cells inside separated nanoclusters (9, 10). Furthermore, we showed that the B cell coreceptors and marker proteins CD19, CXCR4, CD40, and CD20 are part of the IgD-class nanocluster on the B cell surface and that the colocalization is of functional relevance (11). Antigen-dependent activation of B cells resulted in a nanoscale reorganization of the B cell membrane so that CD19 and CD20 were now found in close proximity to the IgM-BCR (9, 12). The formation of the IgM-BCR/CD19 signaling synapse allowed IgM to gain access to the PI-3 kinase signaling pathway promoting the survival and differentiation of B cells. In the present study, we show that CD20 controls the proper nanoscale receptor organization on resting B cells and prevents uncontrolled IgM-BCR/CD19 signaling.

## Results

**Fig. 1.**
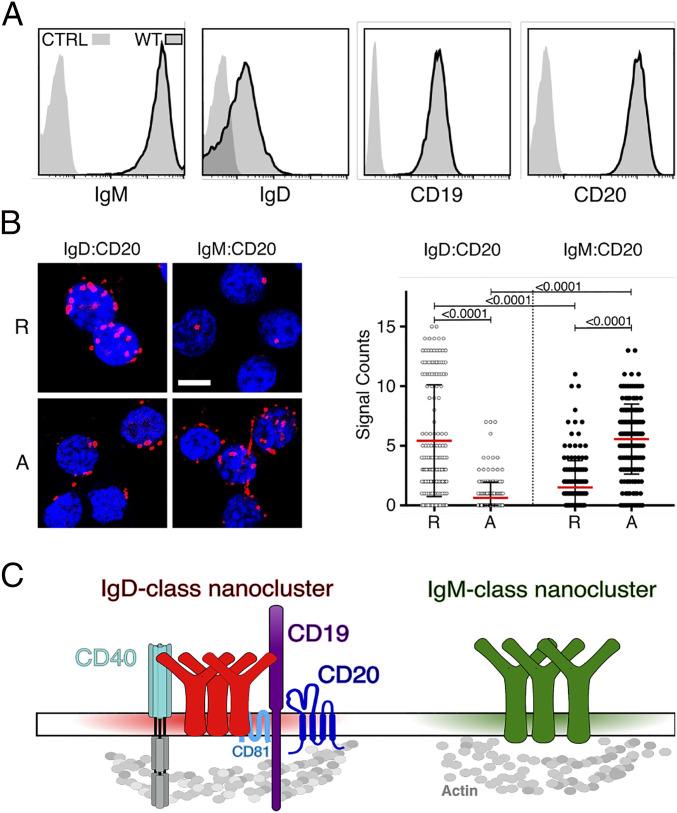
CD20 expression and localization on WT Ramos B cells. (*A*) Flow cytometry analysis showing the expression of IgM-BCR, IgD-BCR, CD19, and CD20 on Ramos WT cells compared to unstained control (CTRL); *n* = 18. (*B*) Fab-PLA study of the proximity of CD20 to either the IgD-BCR or to the IgM-BCR on resting (R), or 5-min pervanadate-activated (A) WT Ramos B cells. Representative microscope images (*Left*) of PLA signals are shown in red and nuclei in blue. (Scale bar, 10 µM.) Scatter dot plot represents the mean (red bar) and SD of PLA signals (Signal Counts); *n* = 3. (*C*) Schematic drawing of the proposed localization of coreceptors and surface marker within IgM and IgD-class protein islands on the surface of human resting B cells.

**Fig. 2.**
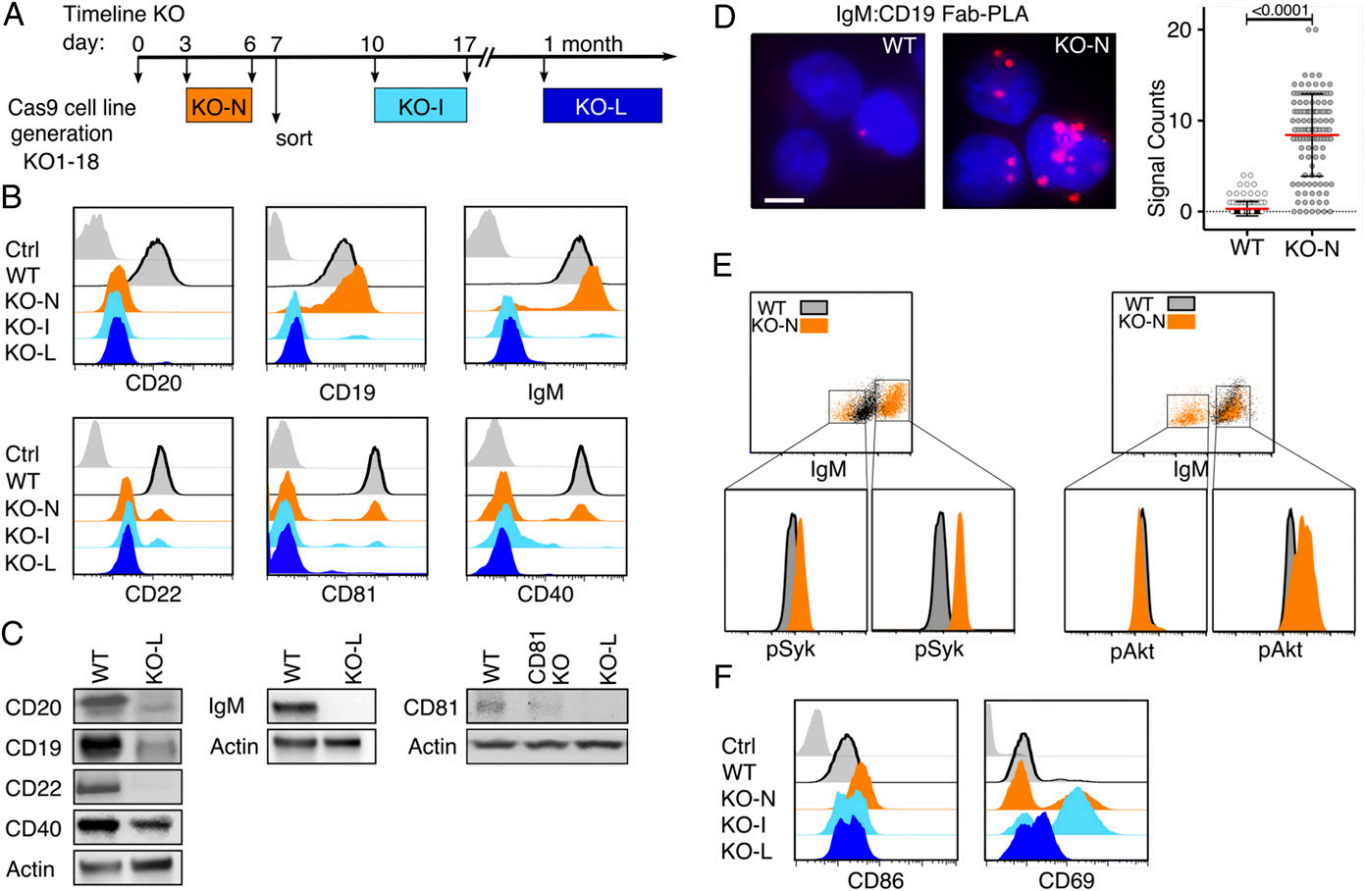
Loss of CD20 results in altered expression of B cell surface markers and transient activation. (*A*) Timeline of developmental states in CD20KO Ramos cell line generation. MS1A4 gene targeting with CRISPR/Cas9 at day 0, developmental stage at days 3 to 6 shown as CD20KO-new (KO-N, orange), at days 10 to 17 shown as CD20KO-intermediates (KO-I, light blue), and after 1 mo shown as CD20KO-late (KO-L, dark blue). (*B*) Flow cytometry analysis showing expression of surface molecules of KO-N, KO-I, and KO-L Ramos cells over time compared to WT and unstained control (gray); *n* = 18. (*C*) Representative Western blot analysis of KO-L Ramos B cell lysates (*Right*) compared to WT (*Left*). Lysates were taken 20 d after *MS4A1* gene targeting; *n* = 14 (*D*) Fab-PLA analysis of IgM-BCR proximity to CD19 in resting WT Ramos B cells compared to the unstimulated KO-N cells 3 d after *MS4A1* gene targeting. (Scale bar: *Left*, 10 µm.) PLA microscope images were quantified as scatter dot plot with mean and SD (*Right*); *n* = 3. (*E*) Intracellular flow cytometry analysis of phosphorylated Akt (pAkt-Ser473) or phosphorylated Syk (pSyk-Tyr525,526) of KO-N cells compared to WT. Gating used to analyze high IgM-BCR expressing KO-N cell population for pSyk or pAkt levels, respectively. (*F*) Flow cytometry analysis showing the over-time expression of the B cell-specific surface activation markers CD86, and CD69 of KO-N compared to WT.

The human Burkitt lymphoma B cell line Ramos carries large amounts of the IgM-BCR on the cell surface, whereas the IgD-BCR is less abundant on these cells (Fig. 1*A*). Ramos B cells also express the coreceptor CD19 and the B cell-specific membrane protein CD20 (13–15). We have previously localized the CD19/CD81 module together with CD20 and CD40 inside an IgD-class specific membrane compartment on the surface of resting B cells (9, 12). To determine the nanoscale organization of the CD20 molecule on the Ramos B cell surface, we used the Fab-based proximity ligation assay (Fab-PLA). Similar to our previous studies on primary B cells, we find CD20 in close proximity to the IgD-BCR on resting Ramos cells whereas after a pervanadate-mediated B cell activation CD20 moves to the IgM-BCR (Fig. 1*B*). Thus, the nanoscale membrane organization of CD20 seems to be similar on Ramos and primary B cells (Fig. 1*C*). Using the CRISPR/Cas9 technology, we targeted exon 3 of the *MS4A1* gene encoding CD20 and disrupted the open reading frame of this gene shortly after the ATG start codon (*SI Appendix*, Fig. S1) (15). The obtained CD20KO Ramos cell lines were classified according to the date of gene targeting as new (KO-N, CD20KO cells in the first week after transfection), intermediate (KO-I, cells in the second to third week), and late (KO-L, cells after 1 mo) cells (Fig. 2*A*). The KO-I and KO-L cells were negatively selected for the loss of CD20 expression by cell sorting. In total, we generated 18 separate CD20KO Ramos cell lines that were analyzed at different time points for the abundance of selected cell surface markers. Surprisingly, the KO-I and KO-L Ramos cells not only lost the surface expression of CD20, but also that of other B cell surface markers such as CD19, CD22, CD81, CD40, and the IgM-BCR (Fig. 2*B*). The altered protein abundance was confirmed by Western blotting analysis of lysates from KO-L cells, which showed a loss, or marked reduction of CD20, CD19, CD22, CD40, IgM, and CD81 proteins in comparison to Ramos wild-type (WT) cells (Fig. 2*C*). Thus, the CRISPR/Cas9-induced CD20 gene deficiency is accompanied by drastic changes of the receptor abundances on the surface of Ramos cells and the loss of the B cell resting stage.

### Loss of CD20 Expression Is Associated with B Cell Activation.

Similar to KO-I and KO-L cells, KO-N Ramos cells showed reduced expression of CD20, CD22, CD81, and CD40; however, CD19 and IgM-BCR levels were increased (Fig. 2*B*). On resting murine and human B cells, CD19 and the IgM-BCR are separated from each other but form a signaling synapse upon B cell activation (9, 16). Interestingly, an increased IgM:CD19 proximity was detected by Fab-PLA on KO-N Ramos cells, indicating that these cells are activated (Fig. 2*D*). Indeed, a phosphor-flow cytometry analysis of WT and KO-N Ramos cells revealed an increased phosphorylation of Syk and Akt in the KO-N population with the highest IgM expression (Fig. 2*E*). Furthermore, KO-N cells up-regulated the B cell activation markers CD86 and CD69 (Fig. 2*F*). Thus, the CD20 gene deletion is associated with a transient B cell activation followed by a loss/down-regulation of several B cell surface markers. Together, these data suggest that on resting B cells CD20 functions as a gatekeeper preventing uncontrolled IgM-BCR/CD19 interaction and signaling.

To learn more about the molecular requirements for the changes of B cell surface expression displayed by the CD20KO Ramos cells, we generated CD20 loss mutants from Ramos CD19KO and from BCR-KO cells, deficient in heavy chain, light chain, and activation-induced deaminase (AID) expression. Unlike KO-L, the CD19/20DKO-L Ramos cells still expressed the IgM-BCR and CD22 (*SI Appendix*, Fig. S2*A*), whereas the BCR/20DKO-L Ramos cells retained expression of the B cell surface marker CD19 and CD22 (*SI Appendix*, Fig. S2*B*).

Together, this analysis suggests that the BCR and CD19 interaction is indispensable for the transient B cell activation and the altered surface expression of CD20KO Ramos B cells.

### Reexpression of CD20 Restores the Resting B Cell Phenotype.

**Fig. 3.**
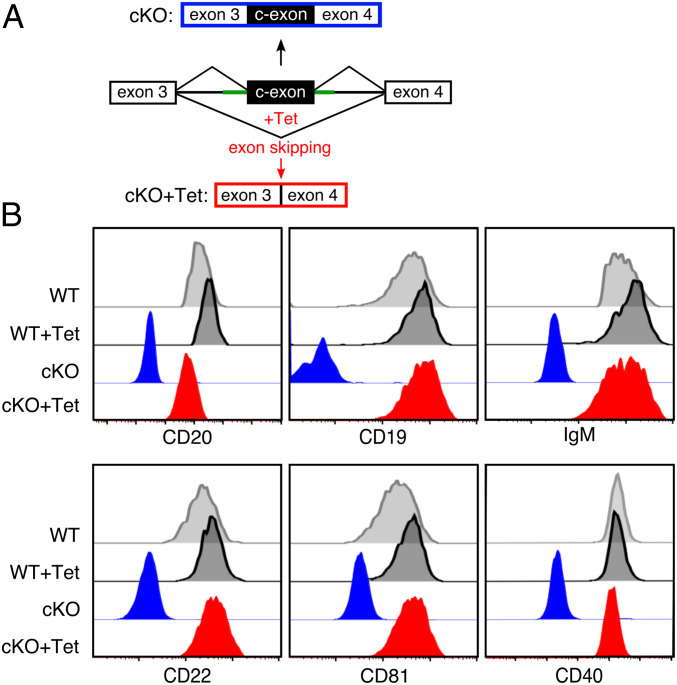
Gain-of-function studies with a conditional CD20KO (cKO). (*A*) Schematic of CRISPR-mediated insertion of an aptamer-controlled exon (c-exon) in between exon 3 and exon 4 of the human *MS4A1* gene generating a CD20 conditional KO (cKO-top). Tetracyclin (Tet) induces c-exon skipping and restores the ORF of the *MS4A1* gene (cKO-Tet; *Bottom*). (*B*) Flow cytometry analysis of the expression of the surface markers CD20, CD19, IgM, CD22, CD81, and CD40 on cKO (blue) or cKO-Tet Ramos cells (red). The untreated (WT) and Tet-treated (Ramos-Tet) Ramos cells are shown as control (gray). The cKO-Tet cells are derived from 30 d posttransfection cKO Ramos B cells treated for 12 h with 6 µM Tet to restore CD20 expression, *n* = 4.

We next asked whether the reexpression of CD20 could restore the expression of cell surface proteins on CD20-deficient Ramos B cells. For this, we established a conditional KO (cKO) of the CD20 encoding *MS4A1* gene (Fig. 3). Using the CRISPR/Cas9 technique, we inserted between exon 3 and exon 4 of the *MS4A1* gene, an aptamer-controlled conditional exon (c-exon) whose incorporation in the processed MS4A1 mRNA results in a stop code, thus rendering this transcript defective (17). Upon exposure of the Ramos cKO cells to tetracycline (Tet), the antibiotic molecule binds to and stabilizes an aptamer whose sequence partly overlaps with the 3′ splice site of the c-exon, thus preventing its incorporation into the CD20 transcript (Fig. 3*A*). This synthetic tetracycline riboswitch enables the successful regulation of CD20 protein levels. Similar to KO-L, the cKO Ramos B cells cultured for more than 20 d did not express CD20 nor CD19, IgM-BCR, CD22, CD81, or CD40 on the B cell surface (Fig. 3*B*). An overnight (o/n) Tet-exposure of the cKO Ramos cells restored the expression of CD20 and that of the other B cell markers on the cell surface. Thus, upon its reexpression, CD20 can resume its gatekeeper function to support the resting state of Ramos B cells.

### RTX Treatment of Naive Human B Cells Terminates the Gatekeeper Function of CD20.

**Fig. 4.**
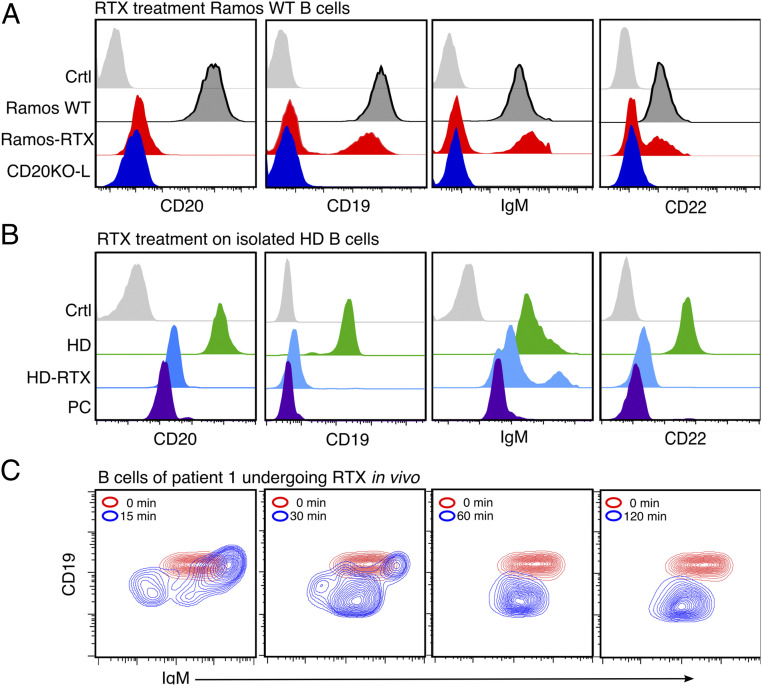
In vitro and in vivo treatment with rituximab (RTX) leads to CD20KO phenotype. (*A*) Flow cytometry analysis after treatment with RTX for 3 d showing the loss of CD19, IgM-BCR, and CD22 on Ramos-RTX compared to CD20KO-L, untreated Ramos-WT, and unstained control (Ctrl); *n* = 3 (*B*) Flow cytometry analysis of negatively selected naive B cells from peripheral blood of a healthy donor were treated with RTX for 60 min (HD-RTX) and compared to untreated (HD), to plasma cells (PCs) of the same donor, or left untreated and unstained (Ctrl); *n* = 3. (*C*) Example of B cells taken from EDTA whole-blood samples of RA patient undergoing RTX treatment after 0, 15, 30, 60, and 120 min. RTX (1 mg/mL) flow rate, 50 mL/h. Flow cytometry staining of CD19^+^/CD27^−^/IgM^+^ selected B cells shows internalization of IgM and CD19; *n* = 3.

CD20 is a prominent target of therapeutic antibodies such as RTX that are used for the depletion of B lymphocytes in autoimmune diseases and B cell neoplasias. To test whether the RTX treatment interferes with the gatekeeper function of CD20, we used flow cytometry to monitor the expression of B cell surface markers on WT Ramos cells (Fig. 4*A*), and healthy donor (HD) derived naive B cells (Fig. 4*B*) before or after a RTX treatment. We extended investigation on the diffuse large B cell lymphoma–germinal center B cell-type (DLBCL-GCB) cell line OCI-LY7 (*SI Appendix*, Fig. S7), which is highly dependent on sustained BCR signaling (18). Interestingly, all RTX-treated cells lost CD20 from the surface and simultaneously down-regulated CD19, CD22, and the IgM-BCR to a similar extent as the KO-L Ramos cells, but primary B cells developed much faster. We suggest that this discrepancy relies on the higher level of membrane proteins in dividing cancer cells compared to a normal protein turnover in primary naive B cells. To further test for the immediate effect of RTX in vivo, we analyzed blood samples taken from patients suffering from RA undergoing RTX treatment. B cells from these patients showed a transient increase of surface IgM-BCR expression already after 15 min, followed by a continuous IgM-BCR down-regulation. CD19 surface expression, which is routinely used as a B cell marker, was almost lost after 30 min of RTX treatment, although B cells were still present and detectable (Fig. 4*C*). A Fab-PLA study showed an increased BCR-IgM:CD19, Igα:pSyk, Igα:pYp110δ, and Igα:pAkt proximity in RTX-treated compared to untreated HD B cells, indicating that the RTX treatment is accompanied by increases of BCR-associated Syk/PI-3kinase signaling and B cell activation (*SI Appendix*, Figs. S3 and S4). In summary, these data show that CD20 is a gatekeeper also for resting naive human B cells and that a CD20 deficiency or RTX treatment terminates B cell dormancy.

### Dynamic Alteration of the Surfaceome of CD20-Deficient B Cells.

To obtain a more global picture of the alteration of protein expression on the surface of CD20-deficient Ramos B cells, we took advantage of the cell surface capture (CSC) technology (19, 20). CSC technology allows for the efficient labeling, identification, and relative quantification of N-glycosylated proteins residing on the cell surface and provides a snapshot of the surfaceome without having to use antibodies to broadly phenotype the B cells (21). The CSC analysis of the 3- to 7-d-old KO-N Ramos cells showed a significant up-regulation of surface markers (3 down vs. 45 up; value of *P* < 0.05) that is consistent with the activated state of these cells (*SI Appendix*, Fig. S5 *A* and *B*). The proteins with higher cell surface abundance include the IgM-BCR and CD19, thereby confirming our previous flow cytometry analysis (Fig. 2*B*). Interestingly, the loss of CD20 on KO-N Ramos cells resulted in the transient, but significant up-regulation of several tetraspanins such as CD37, CD53, CD63, CD82, and TSN3 that could contribute to nanoscale membrane organization as well (22). In contrast to KO-N, the KO-L Ramos cells displayed a reduction in the cell surface abundance of many proteins (123 down vs. 58 up; value of *P* < 0.05), indicating that at a later time point CD20 deficiency is associated with gene silencing (*SI Appendix*, Fig. S5 *C* and *D*). Indeed, the abundance of many B cell markers, such as the BCR, CD19, CD38, CD27, and CD22, was reduced on KO-L Ramos cells. Interestingly, several surface proteins, such as ITGB1, SSR1, LMAN2, CLCN3, STT3A, SEL1L, SSR2, and HSP90B1/ENPL, that were up-regulated on KO-L Ramos cells were also found to be highly abundant on the surface of plasma cells (PCs). This suggests that the loss of CD20 is not only associated with a loss of the resting B cell stage but also an increased differentiation toward the PC stage.

### Plasma Cell Differentiation of CD20-Deficient B Cells.

**Fig. 5.**
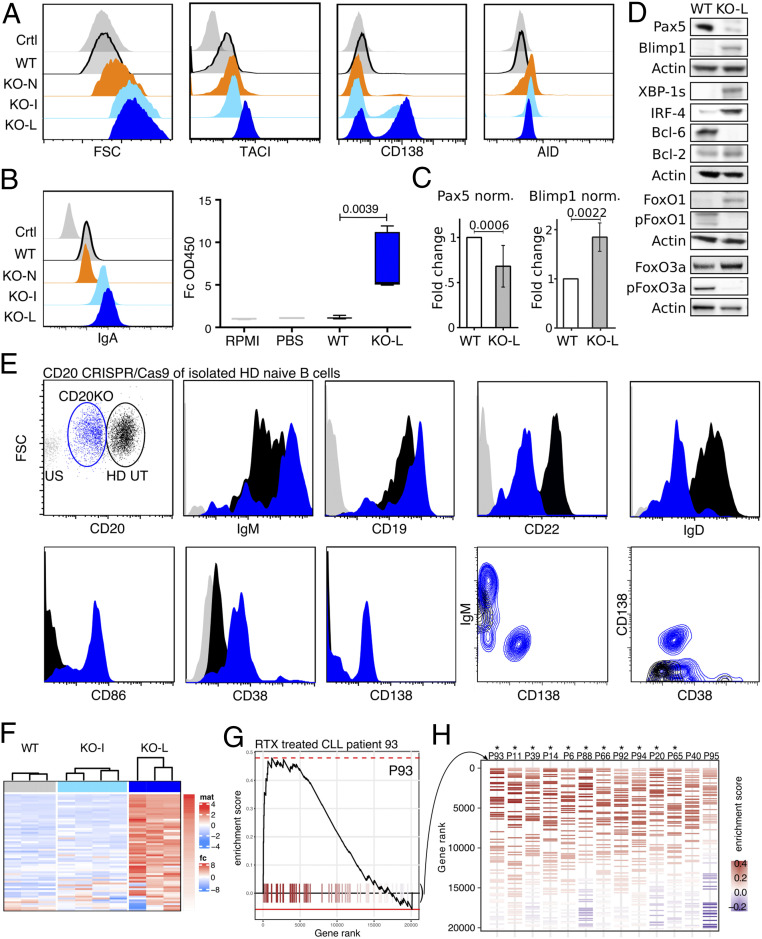
Increased PC differentiation of CD20KO Ramos B cells and primary naive B cells and of CLL B cell relapse after RTX therapy. (*A*) Flow cytometry analysis showing size (FSC-A) and expression of PC markers TACI, CD138, and AID on KO-N, KO-I, and KO-L Ramos B cells compared to WT and unstained control (Ctrl); *n* = 6. (*B*) Expression of surface IgA-BCR (*Left*) and ELISA of IgA secretion (*Right*) of KO-L compared to Ramos WT, PBS, and RPMI control. (*C*) Summarized intracellular flow cytometry analysis of six independently generated CD20KO Ramos B cell lines showing the fold change (FC) of Pax5 and Blimp1 expression of KO-L Ramos B cell lines compared to WT. (*D*) Representative examples of Western blot analysis for B cell differentiation markers of KO-L Ramos B cells compared to WT. Lysates were taken 20 d after induction of CD20KO; *n* = 3. (*E*) Flow cytometry analysis 24 h after CD20CRISPR/Cas9 CD20KO of isolated naive HD B cells. CD20^low^ B cells were gated and shown in blue, compared to transfection control (empty plasmid) in black; *n* = 3. (*F*) PLASMA UP GENES Heatmap, expression of PC differentiation up-regulated genes. The color code indicates the row-wise scaled intensity across the samples. Genes are ranked according to their log2 fold FC in WT (*Left*) vs. KO-L (*Right*); *n* ≥ 3. (*G*) Example of enrichment plot (curve) of PC differentiation up-regulated genes (ticks) in one patient. (*H*) Enrichment barcode illustrating the distribution of PC differentiation up-regulated genes (colored segments) in every individual patient. From GSE37168. Treated patients were ordered from *Left* to *Right* based on their enrichment score, from high to low. Significant enrichment scores are depicted by an asterisk (*).

In culture, with time CD20KO Ramos cells become larger as indicated by the increased forward scatter (FSC) and up-regulated TACI and CD138, both PC markers (Fig. 5*A*). An intracellular fluorescence-activated cell sorting (FACS) analysis showed that CD20KO Ramos cells up-regulate AID at the early KO-N stage at a time point when they display active Syk/PI-3 kinase signaling. Thus, similar to activated B cells participating in a germinal center (GC) reaction, the CD20KO Ramos seem to undergo somatic hypermutation and class switching. Indeed, we found the KO-L Ramos cells to carry IgA on their cell surface and to secrete IgA (Fig. 5*B*). In normal activated B cells, the differentiation to the PC stage is associated with a transcriptional switch involving the down-regulation of the transcription factor PAX5 and up-regulation of Blimp-1. Western blot analysis showed that this is also the case in CD20KO Ramos cells (Fig. 5*C*). In comparison to WT, the KO-L Ramos cells expressed less PAX5 and more Blimp-1. Furthermore, KO-L Ramos cells contained more FoxO1, IRF4, and XBP1s, transcription factors that are associated with B-to-PC differentiation. Another known regulator of PC differentiation is the Blimp-1 suppressor and transcriptional silencer Bcl6, which is highly expressed in GC B cells as well as GC-derived B lymphomas such as Ramos (23–26). Indeed, Bcl6 was expressed in WT Ramos but no longer detectable in the KO-L cells. The PAX5 to Blimp-1 transcriptional switch was not detected in the CD19/20DKO-L and BCR/20DKO-L Ramos cells, indicating that BCR/CD19 signaling and transient B cell activation was required for this in vitro human PC differentiation (*SI Appendix*, Fig. S6 *C* and *D*). To test whether the loss of CD20KO will also lead to activation and expression of PC-related surface receptors in primary B cells, we performed a CRISPR/Cas9-induced CD20KO in isolated HD naive B cells and measured the expression of key surface receptors 24 h later by flow cytometry. The transfected CD20^low^ B cell population showed a similar surface receptor expression profile for the IgM-BCR, CD19, and CD22 as already detected for CD20KO-N Ramos cells and under RTX treatment in RA patients as well. CD20^low^ B cells also decreased the surface abundance of the IgD-BCR and up-regulated expression of CD86, CD38, and CD138 (Fig. 5*E* and *SI Appendix*, Fig. S4*B*). We concluded that if the loss of CD20, be it induced by CRISPR/Cas9 KO or after RTX therapy, is accompanied by an increased PC differentiation, then we should identify PC-specific genes after RTX therapy of B cell malignancies in vivo. For this, we analyzed the transcriptome profile of B cells from CLL patients (27) that have relapsed after a combined RTX treatment (RTX ± fludarabine) for the expression of PC-specific genes in a gene set enrichment analysis (GSEA) and compared this gene set with Ramos WT and CD20KO-L cells (Fig. 5*F*). Interestingly, the samples from 11 of 13 CLL patients showed a significantly increased expression of PC-specific genes after combined RTX treatment, confirming our results that CD20-derived B cell activation can induce activation of the PC transcription program (Fig. 5 *G* and *H*) (Datasets S1 and S2).

### Distinct Alterations of Cell Metabolism during Ramos Plasma Cell Development.

**Fig. 6.**
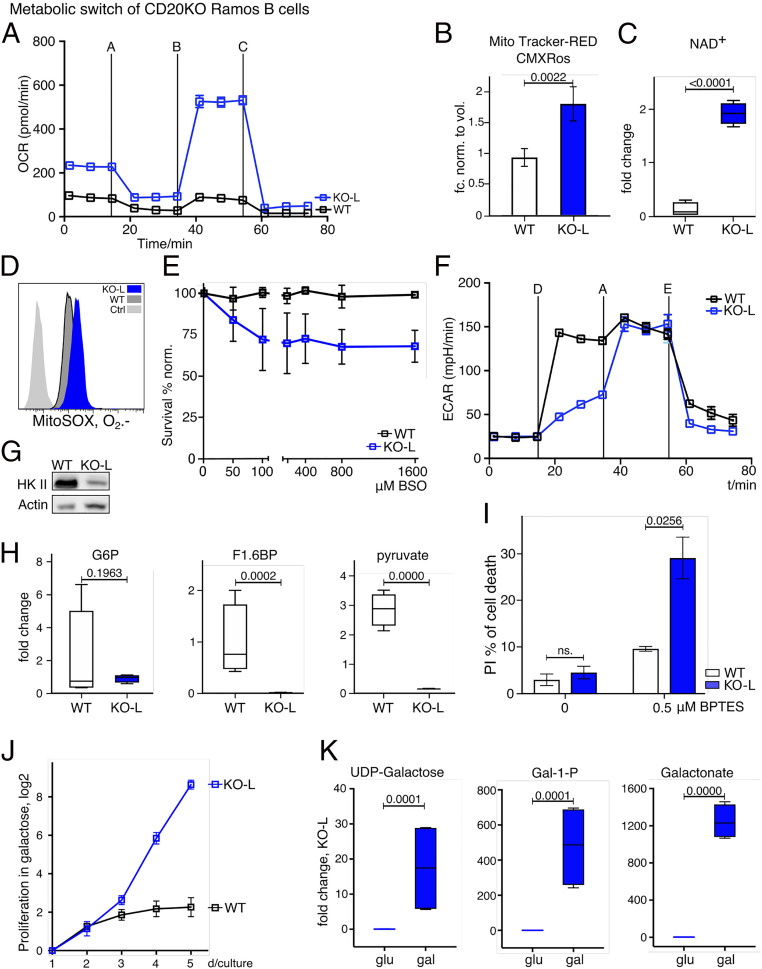
Metabolic switch of CD20KO Ramos B cells. (*A*) Oxygen consumption rate (OCR) of KO-L compared to WT Ramos cells. Metabolite flux analysis was performed in triplicate and is displayed as mean ± SD. One, out of three experiments, is shown. Used inhibitors: A = oligomycin; B = FCCP; C = rotenone plus antimycin. (*B*) Mitochondrial mass staining with Mito Tracker Red-CMXRos in KO-L compared to WT Ramos cells and normalized to volume of WT Ramos cells; *n* = 4. (*C*) NAD^+^ levels of KO-L compared to WT Ramos cells determined with untargeted metabolomic profiling; *n* = 3. (*D*) Mitochondrial superoxide levels stained with MitoSox Red. KO-L cells, WT Ramos cells, and unstained control (Ctrl) are shown; *n* = 4. (*E*) Survival rates (propidium iodide [PI]-negative cells) of KO-L and WT Ramos cells after buthionine sulfoximine (BSO) titration. The experiment was performed three times and mean MFI values were normalized to day 0. (*F*) Extracellular acidification rate (ECAR) of KO-L and WT Ramos cells as a measure of glycolysis is shown. The measurement was performed in technical triplicates and is displayed as mean ± SD. One, out of three independent experiments, is shown. D = glucose, A = oligomycin, and E = 2DG. (*G*) Representative Western blot analysis of hexokinase II (HK II) protein levels of KO-L and WT Ramos cells. Lysates were taken 20 d after induction of CD20 KO, and data are representative for at least three independently generated CD20 KO Ramos B cell lines. (*H*) Levels of the glycolytic intermediates glucose 6-phosphate (G6P), fructose 6-phosphate (F1,6BP), and pyruvate as determined by untargeted metabolomic profiling are shown; *n* = 3. (*I*) Cell death as determined by PI staining after treatment with BPTES; *n* = 3. (*J*) Proliferation index determined with MFI of CellTrace staining per day of KO-L cells cultivated in galactose compared to WT Ramos cells; *n* = 3. (*K*) Levels of UDP-galactose, galactose-1-phosphate (G 1-P), and galactonate as determined by untargeted metabolomic; *n* = 4.

PCs are large cells with an extended endoplasmic reticulum and a high rate of antibody production. PCs thus have a higher demand for energy and biosynthetic precursor molecules than B lymphocytes (28, 29). To characterize the metabolic program of WT and KO-L Ramos cells, we performed metabolic flux experiments and untargeted metabolomics analyses. We found the abundance of 117 biochemicals to be significantly increased and the abundance of 214 biochemicals to be significantly decreased in KO-L Ramos cells in comparison to WT cells. Analysis of the oxygen consumption rate (OCR) showed that KO-L cells consume more oxygen in the basal state and possess a higher spare respiratory capacity than WT Ramos cells (Fig. 6*A* and Dataset S3).

Nicotine amid adenine dinucleotide (NAD+) is a crucial cofactor for enzymes in several metabolic pathways and high levels of NAD+ have been suggested to favor mitochondrial biogenesis and function (30). We found mitochondrial mass (Fig. 6*B*) and NAD+ levels (Fig. 6*C*) to be significantly increased in KO-L Ramos cells in comparison to WT cells. Since reactive oxygen species (ROS) are a natural by-product of respiratory activity, we hypothesized that ROS production might be altered in KO-L Ramos cells. Indeed, we found O_2_^.−^ levels to be increased (Fig. 6*D*) in KO-L Ramos cells. Moreover, we found the glutathione-cysteine ligase inhibitor buthionine sulfoximine (BSO) to induce cell death of KO-L Ramos cells, and less so in WT cells (Fig. 6*E*). Glutathione-cysteine ligase is an important component of the glutathione-dependent ROS scavenging pathway and protects cells from ROS induced cellular damage. Our results thus suggest that KO-L Ramos cells are exposed to increased levels of ROS and that adaptations to oxidative stress are crucial for their survival.

We next compared the glycolytic activity of WT and KO-L Ramos cells by measuring the extracellular acidification rate (ECAR). As expected for a Burkitt lymphoma, WT Ramos cells showed robust increase of ECAR after receiving glucose (Fig. 6*F*). Upon oligomycin treatment, which is an inhibitor of mitochondrial ATPase, ECAR was further increased to compensate for the loss of mitochondria-derived ATP. In contrast to WT cells, KO-L cells showed low lactate production upon receiving glucose, yet ECAR levels were similar to those seen in WT after oligomycin treatment (Fig. 6*F*). This suggests that, although KO-L cells are capable of converting glucose to lactate, this metabolic pathway is not favored under normal conditions. Consistent with their reduced glycolytic rates, the KO-L cells showed reduced expression of hexokinase II (HKII), the enzyme mediating the first step of glycolysis (Fig. 6*G*). Furthermore, we found the levels of glycolytic intermediates such as glucose 6-phosphate, fructose 6-phosphate, and pyruvate to be decreased in KO-L cells (Fig. 6*H*). Taken together, these data suggest that glucose is not the primary source of energy in KO-L Ramos cells.

To assess whether alternative carbon sources play a more prominent role in KO-L Ramos cells, we treated the cells with Bis-2-(5-phenylacetamido-1,3,4-thiadiazol-2-yl)-ethyl sulfide (BPTES), an inhibitor of glutaminolysis. We found that while BPTES treatment induced cell death of KO-L cells, WT Ramos cells were less affected (Fig. 6*I*). Furthermore, unlike WT Ramos, KO-L cells can be cultured in a medium where glucose is replaced by galactose (Fig. 6*J*). KO-L cells are able to process galactose intracellularly and use it as an alternative carbon source (Fig. 6*K*). Unlike glucose, galactose needs to be catabolized in the mitochondria to provide cells with energy. Thus, our results demonstrate that KO-L Ramos cells can survive entirely without glycolysis-derived ATP.

In summary, our metabolic analysis demonstrates that the loss of CD20 results in profound metabolic alterations, which in many instances mirror the phenotype of PCs/plasma blasts (31, 32).

## Discussion

Macfarlane Burnet’s clonal selection theory provided the foundation for the explanation of adaptive immunity. The proposed selection process requires a strict separation between resting and activated B lymphocytes. We show here that CD20 is a gatekeeper of the resting B cell state. On resting mature B lymphocytes, the IgM-BCR and the IgD-BCR reside on the cell surface inside separated nanodomains with distinct lipid and protein compositions. Together with CD19, CD20 is a component of the IgD-class nanodomains. Upon antigen-dependent B cell activation, this nanoscale receptor organization is altered and CD19 together with the IgM-BCR forms a signaling synapse. We find that the exposure to anti-CD20 antibodies in vitro and the loss of CD20 also results in IgM/CD19 synapse formation, association to Syk/PI-3 kinase signaling and transient B cell activation by itself.

CD20 deficiency in mice and humans has been associated with reduced B cell signaling and T cell-independent immune responses rather than increased BCR signaling (33, 34). The reason why murine CD20KO has a rather mild phenotype is due to not well-studied compensation mechanisms including transcriptional adaptations of a B cell population under a stringent developmental selection process in rather artificial inbred mouse strains (35, 36). In this respect, our study removing a key regulator such as CD20 at the mature (and not a developing) B cell stage (in human B cell tumors and normal human B cells) is more physiological and suitable to reveal the proper function of a gene product supporting the resting state of mature B cells. With the CRISPR/Cas9 technique, it is now feasible to establish a CD20 deficiency at the mature B cell stage without interference of the B cell selection process and to combine several gene KO for a gene function analysis. In this way, we could show that the transient B cell activation is associated with PC differentiation of all our Ramos cell lines. With an independent dataset, a GSEA based on transcriptomic profiling revealed PC-specific genes up-regulated after combined RTX treatment in the transcriptome of about 85% of the investigated patients (27). Disruption of the nanoscale organization of CD20 followed by development of PCs could explain the results of a study on immune thrombocytopenia patients after RTX medication, which suggests that B cell depletion generates a milieu that promotes the differentiation and settlement of long-lived PCs in the spleen (37, 38). It is known that PCs improve metabolic flexibility to secure function and prolonged survival (29, 38). Our analysis of the metabolic program of KO-L Ramos cells allowed us to identify metabolic vulnerabilities in these cells. Our finding that CD20KO Ramos cells switch from glycolysis to oxidative phosphorylation and from glucose to glutamine usage is in line with previous studies of PC metabolism (29, 30). Equally, our finding that glucose does not induce high levels of lactate secretion in CD20KO Ramos cells is consistent with published studies showing that PCs primarily use glucose for antibody glycosylation (39). Importantly, the insight obtained from this metabolic profiling enabled us to identify BPTES and BSO as inhibitors reducing the viability of KO-L Ramos cells, and these compounds provide a rationale to target these metabolic pathways in PC pathologies. Whether the presence of CD20-negative cancer B cells could affect the generation of new PCs in response to infection or vaccination is another outstanding issue (40, 41). Our finding of PC characteristics and adaptations in CD20-negative cancer B cells opens possibilities for patients that have relapsed after RTX medication. These patients may benefit from a treatment tailored to make use of the specific biological properties of PCs.

## Materials and Methods

### Primary Human Naive B Cells.

Primary naive B cells were obtained from fresh buffy coats provided by the Institute for Transfusion Medicine and Gene Therapy, ITG Freiburg. Peripheral blood mononuclear cells were separated by Ficoll gradient centrifugation of whole blood and negatively selected using EasySep Human Naive B Cell Isolation kit (StemCell). Prior to the experiments, primary naive B cells were controlled for purity and rested overnight. The study was approved by the Institutional Review Board of the University of Freiburg (ethical vote nos. 507/16 and 336/16). Additional details can be found in *SI Appendix*, *Methods*.

### Cell Culture.

The human Burkitt lymphoma B cell-line Ramos was obtained from American Type Culture Collection (ATCC), Ramos (ATCC; catalog #CRL-1923, RRID: CVCL 1646), the DLBCL-GCB OCI-Ly7 was kindly provided by John Apgar (Sanford Burnham Prebys Medical Discovery Institute, La Jolla, CA). For glucose vs. galactose metabolism analysis, Ramos B cells were cultured in RPMI medium 1640 without glucose (Gibco), supplemented with 11 mM sterile filtered glucose or galactose, respectively, 5% dialyzed FCS (Thermo Fisher), 10 units/mL penicillin/streptomycin (Gibco), 20 mM Hepes (Gibco), and 50 mM β‐mercaptoethanol (Sigma). Additional details can be found in *SI Appendix*, *Methods*.

### CRISPR/Cas9 Knockout.

CRISPR/Cas9 deletion was either carried out using the Neon Transfection System (Invitrogen) to either deliver the human MS4A1 KO plasmids (Santa Cruz) into the cells or the ribonucleoprotein according to the genome editing method of Integrated DNA Technology (IDT) (42). For plasmid delivery, 1.1 × 10^6^ cells were resuspended with 110 μL of transfection medium containing 20 mM Hepes (Gibco) and 1.25% DMSO (Sigma) in RPMI medium together with 4 μg of KO plasmid and subjected to Neon Transfection System. Electroporation was performed in 100-µL NEON tips at 1,350 V, 30 ms, single pulse in recovery medium. Specificity of the *MS4A1* gene KO was controlled in parallel by transfection of a control plasmid (Santa Cruz) or by transfection of a negligible protein (p62). Inactivation of the target gene was verified by flow cytometry and/or Western blotting. Additional details can be found in *SI Appendix*, *Methods* and Dataset S4.

### Flow Cytometry Analysis.

For surface staining, 1–20 × 10^5^ cells were stained on ice, washed twice, and then measured with a FACS Gallios (Beckman Coulter). For intracellular staining, the cells were fixed with 4% PFA and permeabilized with 0.5% saponine. Data were exported in FCS‐3.0 format and analyzed with FlowJo software (TreeStar). A Bio-Rad S3e cell sorter was used to select CD20-negative cell populations. Additional details can be found in *SI Appendix*, *Methods* and Dataset S4.

### Lymphocyte and B Cell Subpopulations Phenotyping.

Phenotyping of T, B, and NK cells within the lymphocyte population was performed by a whole blood staining lyse-no-wash protocol (Optilyse B; Beckman Coulter). To determine B cell subpopulations, nine color flow cytometry with fluorochrome-conjugated antibodies was performed. Gating strategy is in *SI Appendix*, Fig. S3. Cells were measured by flow cytometry (Navios; Beckman Coulter) and analyzed using Kaluza Software 1.5a (Beckman Coulter). Further details are provided in *SI Appendix*, *Methods* and Dataset S4.

### Cell Survival.

Cell survival was determined by analyzing forward/sideward scatter properties of cells and simultaneous staining with either propidium iodide (PI), or 7-amino-actinomycin D eFluor670 dye, and LIVE/DEAD fixable violet stain (Thermo Fisher), following the manufacturer’s instructions. Cell survival was continuously monitored in all experiments.

### Cell Proliferation Assay.

The cell proliferation assay was performed using CellTrace Proliferation Kit (Thermo Fisher). Cells were stained according to the manufacturer's protocol, and the mean fluorescence intensity (MFI) was measured. Further readings were followed daily. MFI values were used to calculate the proliferation index (43). Details are in *SI Appendix*, *Methods*.

### Western Blotting.

Cells were collected and immediately lysed in 2× Laemmli or RIPA buffer. Equal amounts of cleared lysates were subjected to sodium dodecyl sulfate–polyacrylamide gel electrophoresis on 10%, 12%, or gradient (10–15%) mini precast gels (7bioscience) and to subsequent immunoblotting on polyvinylidene difluoride membrane (GE Healthcare Amersham) and blocked with 5% BSA in PBS and 0.1% Tween 20. A horseradish peroxidase-conjugated goat anti-rabbit or goat anti-mouse antibody was used as secondary antibody before detection with ECL chemiluminescent substrate (Bio-Rad). Lysates were taken from independently generated Ramos CD20KO B cell lines; *n* > 3. Details of primary antibodies are shown in Dataset S4.

### CRISPR Riboswitch Design and cKO Ramos Cell Generation.

The CRISPR riboswitch was designed based on a published exon-skipping RNA device (17). A minimized portion of the original, synthetic construct including part of the 5′ intron, the 3′ splice site, a tetracycline aptamer, the poised exon, the 5′ splice site, and part of the 3′ intron was targeted to intronic sequences of the human CD20 gene. The tetracycline aptamers C1 and C2 used here correspond to those reported in the M1 and M2 minigene constructs (17). The intronic CRISPR guides were designed to avoid regulatory elements using the University of California, Santa Cruz, Genome Browser. The Alt-R Cas9 ribonucleoprotein complexes were assembled as mentioned above (42). The riboswitch was amplified by PCR (CloneAmp; Takara; according to the manufacturer’s instructions) verified by agarose gel, and cleaned using a PCR purification kit. The HDR templates were mixed at fourfold molar excess with a single-strand DNA binding protein (ET SSB; NEB; M2401S) and heated at 95 °C for 10 min to achieve single-stranded donor oligonucleotides bound to ET SSB. Two picomoles of HDR template were combined with the RNP-enhancer-cell mixture and subjected to Neon Transfection System as described above. Further details are listed in *SI Appendix*, *Methods* and Dataset S4.

### Surfaceome Analysis Using CSC.

CSC analysis was performed as previously described (19). More detailed information about the strategy used can be found in *SI Appendix*, *Methods* and Dataset S4.

### PLA.

For Fab‐PLA, the PLA-probes were prepared as previously described (44). F(ab)-fragments were prepared from the corresponding antibodies using the Pierce Fab Micro Preparation Kit (Thermo Fisher). After buffer exchange (Zeba spin desalting columns; Thermo Fisher), F(ab)-fragments were coupled with PLA probemaker (Sigma‐Aldrich). The cells were activated with 1 mM freshly prepared pervanadate or treated with RTX and then fixed for 20 min with 4% paraformaldehyde. Details about PLA-probes are provided in *SI Appendix*, *Methods* and Dataset S4.

### Imaging, Image Analysis, and Data Processing.

All microscope images were acquired using Leica DMi8 microscope equipped with a 63× oil immersion objective lens and analyzed with CellProfiler 3.0.0 and Prism software (GraphPad). Details about PLA-imaging and analysis are provided in *SI Appendix*, *Methods* and Dataset S4.

### Statistical Analysis.

Nonparametric Mann–Whitney *U* test was used for all experiments shown except metabolomics data, which was performed by Metabolon, Inc., using Welch’s two-sample *t* test to determine statistical significance.

### Enzyme-Linked Immunosorbent Assay of IgA Secretion.

Quantification of total IgA antibody secretion was measured by sandwich enzyme-linked immunosorbent assay (ELISA) (Thermo Scientific) with minor modifications. In brief, after centrifugation, media aliquots of three serial dilutions (*n* = 3) of culture supernatants of KO-L and WT Ramos cells were added to 96-well plates coated with 10 µg/mL human anti-lambda specific antibody and kept o/n at 4 °C to calculate maximal effective concentration. RPMI and pure PBS served as a control. After extensive washes with wash buffer containing PBS, supplemented with 0.05% Tween 20 and 0.01% NaN_3_, nonspecific binding was blocked using PBS, supplemented with 1% BSA and 0.01% NaN_3_. Binding was revealed using biotinylated anti-IgA secondary antibody followed by streptavidin–horseradish peroxidase, developed with 3,3′,5,5′-tetramethylbenzidine (TMB), and detected with ELISA microplate reader before normalization. Independently generated Ramos CD20KO-L cell lines were chosen for three repetitions. Details are in Dataset S4.

### Inhibitors.

For inhibition, Ramos cells were treated with BPTES (final concentration of 0.5 µM) or BSO (final concentration 0 to 1,600 μM; all Selleck-Chemicals). Inhibitors were diluted in RPMI culture medium, and treatment was performed as described in the manufacturer’s protocol.

### RTX Treatment.

RTX was kindly provided by F. Hoffmann-La Roche-AG. For Ramos cell treatment, RTX was diluted to a final concentration of 10 µg/mL and performed at least three times. Simultaneous staining of RTX and anti-human CD20 antibody was controlled (*SI Appendix*, Fig. S9).

### Transcriptome Analysis.

Massive analyses of cDNA ends (MACE-Seq) was performed by GenXPro GmbH in Frankfurt am Main using the MACE-Seq kit according to the manual of the manufacturers (45). Briefly, cDNA, generated with barcoded poly-A primers during reverse transcription, was fragmented and a second adapter was ligated. Competitive amplification was used to produce a library that was sequenced on an Illumina NextSeq500 machine. The reads were cleaned from adapter-residues and homopolymer stretches and annotated to the human genome (HG19) and read counts per gene were quantified. Differential gene expression was determined using the limma R package (46) for *P* value and FDR calculation (technical replicates of Ramos WT, *n* = 3; biological replicates of independently generated CD20KO cell lines: KO-I, *n* = 4, and KO-L, *n* = 5).

### Gene Expression Omnibus Data.

Microarray gene expression raw data from 13 CLL patients treated with RTX (27) were downloaded from Gene Expression Omnibus (GEO) (GSE37168). After RMA normalization, single-sample enrichment of PC differentiation up-regulated genes was performed using the fgsea R package (47) on the ranked after vs. before relapse log FC. Statistical significance of the enrichment score was assessed from an empirical null distribution using 1,000 permutations where a value of *P* < 0.05 was considered as significant (listed in Dataset S2). The PC-specific gene set is listed in Dataset S1. Details about GEO data are provided in *SI Appendix*, *Methods*.

### Detection of Mitochondrial Mass.

To stain for mitochondrial mass, 10^5^ cells were stained in 100 µL of 60 nM Mito Tracker-RedCMXRos (Thermo Fisher) in Ramos medium for 30 min at 37 °C. Cells were washed twice before measurement. Cells were analyzed using a flow cytometer Gallios (Beckman Coulter). Data were normalized according to cell volume. Three independent experiments have been performed.

### Detection of Mitochondrial ROS.

Detection of superoxide in mitochondria was performed with MitoSOX-Red mitochondrial superoxide indicator (Molecular Probes) according to the manufacturer’s protocol. In brief, 10^5^ Ramos cells were loaded with 1 mL of reagent working solution (5 µM MitoSOX in HBSS-buffer) and incubated in the dark for 10 min in at 37 °C. The cells were washed three times with warm buffer and immediately subjected to imaging (*n* = 3).

### Metabolomic Analysis.

For metabolomic profiling, cells were washed with PBS and snap frozen in liquid nitrogen. Samples were processed and analyzed by Metabolon. The extracts were divided into five fractions: two for analysis by two separate reverse phases (RP)/UPLC-MS/MS methods with positive ion mode electrospray ionization (ESI), one for analysis by RP/UPLC-MS/MS with negative ion mode ESI, one for analysis by hydrophilic interaction chromatography/UPLC-MS/MS with negative ion mode ESI, and one sample reserved for backup. Raw data were extracted and compounds were peak identified by Metabolon. Missing values were imputed with the observed minimum after normalization. Global metabolic profiles were determined using UPLC-MS/MS (Metabolon). Shown are cell count normalized results for NAD^+^, G6P, F1.6BP, pyruvate, UDP-galactose, Gal-1-P, galactonate. The raw data obtained from our metabolome studies are listed in Dataset S3.

### Metabolomic Flux Analysis.

OCR and ECAR were measured using Seahorse flux technology. Experiments were performed as described before (48). Detailed protocols are included in *SI Appendix*, *Methods*.

Further materials are provided in Dataset S4.

## Supplementary Material

Supplementary File

Supplementary File

Supplementary File

Supplementary File

Supplementary File

## Data Availability

All study data are included in the article and/or supporting information.
