# Supplementary material for: CD20 as a gatekeeper of the resting state of human B cells
Source: Proc Natl Acad Sci U S A. 2021 Feb 9;118(7):e2021342118. doi: 10.1073/pnas.2021342118 (PMC7896350; doi:10.1073/pnas.2021342118)
Supplement: Supplementary File [file pnas.2021342118.sapp.pdf]

PNAS
www.pnas.org

Supplementary Information for

CD20 as a gatekeeper of the resting state of human B cells

Kathrin Kläsener[1,2], Julia Jellusova[1,2], Geoffroy Andrieux[3,4], Ulrich Salzer[5], Chiara Böhler[1,2], Sebastian N. Steiner[6,7], Jonas B. Albinus[6,7], Marco Cavallari[1,2], Beatrix Süß[8], Reinhard E. Voll[5], Melanie Boerries[3,4,9], Bernd Wollscheid[6,7] and Michael Reth[1,2*]

Correspondence to: michael.reth@bioss.uni-freiburg.de

This PDF file includes:

Supplementary text
Figures S1 to S9
Legends for Datasets S1 to S4
SI References

Other supplementary materials for this manuscript include the following:

Datasets S1 to S4

Further Methods

Primary human naïve B cells

Prior to the experiments, primary naïve B cells were controlled for purity and rested overnight in complete RPMI 1640 supplemented with 5 % FCS, 10 units/ml penicillin/streptomycin (Gibco), 20 mM HEPES (Gibco), and 50 mM β-mercaptoethanol (Sigma) at 37°C with 5% CO_2 before subjected to experiment. Experiments with primary human B cells were repeated at least three times.

Cell culture

All cells were cultured in RPMI 1640 medium (Gibco), supplemented with 5% (Ramos) or 10% (OCI-Ly7) heat-inactivated FCS (Biochrom), 10 units/mL penicillin/streptomycin (Gibco), 20 mM HEPES (Gibco), and 50 mM β-mercaptoethanol (Sigma) at 37°C with 5% CO_2.

CRISPR/Cas9 knock out

For CRISPR/Cas9 KO 1.1×10^6 cells were resuspended with 110 µL transfection medium containing 20 mM HEPES (Gibco) and 1.25% DMSO (Sigma) in RPMI medium and then mixed together with 4 µg of KO plasmid. The cells were then subjected to Neon Transfection System. Electroporation was performed in 100 µL NEON tips at 1350 V, 30 ms and a single pulse and recovered at 37°C and 5% CO_2 without antibiotics for one day and then cultured in complete RPMI-medium. For the IDT CRISPR-Cas9 approach equimolar amounts of Alt-R CRISPR-Cas9 crRNA and tracrRNA were annealed in IDTE buffer, combined with Cas9 endonuclease before complex formation with RNP. One million cells were then subjected to Neon Transfection System. Electroporation was performed in 10 µL NEON tips at 1350 V, with a single 30 ms pulse. The transfected cells were cultured at 37°C and 5% CO_2 in complete RPMI medium.

Flow cytometry analysis

For surface staining, $1–20 \times 10^5$ cells were stained with antibodies in PBS supplemented with 0.5% BSA and 0.05% NaN_3 on ice for 20 min, washed twice and then measured with a FACS Gallios (Beckman Coulter) For intracellular staining, the cells were fixed with 4% PFA, washed twice in PBS and permeabilized with 0.5% BSA containing 0.5% saponine for 60 min at room temperature. Data were exported in FCS-3.0 format and analyzed with FlowJo software (TreeStar). A BIO-RAD S3e cell sorter was used to select CD20-negative cell populations. Flow cytometry analysis were performed more than three times from each of the eighteen independently generated CD20KO Ramos B cell lines.

Surfaceome analysis using Cell Surface Capture technology (CSC)

For surfaceome screening 50 million cells per replicate of Ramos WT, CD20KO-N and CD20KO-L were harvested and washed with ice-cold PBS. Deamidated peptides derived from N-glycosylated cell surface-residing receptors were analyzed on a Q Exactive plus

HF mass spectrometer (QE-HF MS) Thermo Scientific) coupled to an EASY-nLC 1200 instrument. The QE-HF MS was operated in positive ion mode and peptides were separated by reverse-phase chromatography on a 15 cm column in-house packed with ReproSil-Pur 120A C18-AQ 1.9 µm (Dr. Maisch GmbH), primed with 100% buffer A (99% H_2O, 01% formic acid) and with a 70-minute gradient from 6 to 44% buffer B (99.9% acetonitrile, 0.1% formic acid) prior to injection. Mass spectrometry data were acquired in data-dependent acquisition (DDA) mode (TOP10). MS1 scans were acquired with 60,000 resolution, AGC target of 3×10^6, IT of 15 ms and followed by high-energy collision dissociation (HCD) at 28%. MS2 scans were recorded with 15,000 resolution, AGC target of 1×10^5 and IT of 110 ms. Raw files were analyzed using the Trans-Proteomic Pipeline (v4.6.2) by searching with COMET (v27.0) against Uniprot KB (Swiss-Prot, Homo sapiens, retrieved April 2018). Carbamidomethylation was set as a fixed modification for cysteine, oxidation of methionine and deamidation of arginine were set as variable modifications. Peptide feature intensities of identified peptides with FDR $\leq$ 1%, presence of consensus NXS/T sequence and deamidation (+0.98 Da) at asparagines were extracted with Progenesis QI (v4.0, Nonlinear Dynamics) for label-free quantification. Statistical analysis was performed with MSstats (v 3.8.6) using iRT peptides to normalize across runs. Significance of differentially regulated proteins was determined by the threshold $|\text{fold-change}| > 1.5$ and $p\text{-value} < 0.05$ of a two-sided t-test with the appropriate degrees of freedom. Benjamini-Hochberg method was used to account for multiple testing. Protein abundance changes and significance was visualized in volcano plots using R. Voronoi treemaps were generated using the FoamTree tool (https://carrotsearch.com/foamtree/). Hierarchical classification was created based on the functional annotation of human plasma membrane proteins (Almén et al., 2009). CSC experiments were performed in triplicates per condition. Link for acquired raw data is deposited to PRIDE and with MS experiment details shown in **Dataset S4.** The MS data (ID:PXD019874) is publicly available at PRIDE and can be accessed at . ftp://massive.ucsd.edu/MSV000085605

Lymphocyte and B-cell subpopulations phenotyping

Phenotyping of T-, B- and NK cells within the lymphocyte population was performed by a whole blood staining lyse-no-wash protocol (Optilyse B, Beckman-Coulter) using six colour flow cytometry with fluorochrome-conjugated antibodies as listed in **Dataset S4** antibodies. Analysis of B-cell subpopulations was performed on ice with a bulk lysis specimen of EDTA anticoagulated whole blood treated with ammonium chloride lysis buffer to lyse erythrocytes. After washing, a lyse-wash protocol (Optilyse B, Beckman-Coulter) was performed. To determine B-cell subpopulations, nine colour flow cytometry with fluorochrome-conjugated antibodies was performed. Gating strategy in **Fig. S4**. Antibody labelled cells were analyzed by flow cytometry (Navios; Beckman Coulter). Flow cytometric data analysis was performed with the help of Kaluza Software 1.5a (Beckman Coulter).

Cell proliferation assay

The cell proliferation assay was performed using CellTrace Proliferation Kit (ThermoFisher) according to the manufacturer's protocol. In brief, one million cells were

washed with PBS and stained in a 1/1000 dilution of the stock solution for 20 min at 37°C protected from light. Cells were washed twice with complete supplemented RPMI. After 10 min the mean fluorescence intensity (MFI) was measured. Further readings were followed daily.

CRISPR riboswitch design and cKO Ramos cell generation

Electroporation was performed in 10 µL NEON tips at 1450 V with a single 20 ms pulse.

Proximity ligation assay (PLA)

For Fab-PLA, F(ab)- fragments were prepared from the corresponding antibodies using the Pierce Fab Micro Preparation Kit (Thermo Fisher) according to the manufacturer's protocol. In brief, after buffer exchange (Zeba™ spin desalting columns, Thermo Fisher) F(ab)-fragments were coupled with PLA probemaker Plus or Minus oligonucleotides according to the manufacturer's protocol (Sigma-Aldrich) to generate Fab-PLA probes. For *in situ* PLA experiments, the cells were allowed to attach to polytetrafluoroethylene (PTFE)-coated slides (ThermoFisher) for 30 min at 37°C. Depending on the experiment, the cells were activated with 1mM freshly prepared pervanadate or treated with RTX and then fixed for 20 min with 4% paraformaldehyde in PBS. PLA experiments were repeated at least three times, each in two technical replicates.

Imaging, Image analysis, and Data processing

All microscope images were acquired using Leica DMi8 microscope equipped with a 63× oil immersion objective lens. For each sample, several images of at least 1000 cells were imaged from randomly chosen regions. All recorded images were analyzed with CellProfiler 3.0.0. Raw data produced by CellProfiler were exported to Prism software (GraphPad, La Jolla, CA). The mean PLA signal count per cell was calculated from the corresponding images and presented as scatter dot plots with mean and standard deviation (SD).

GEO data

By walking down the ranked list of genes, the enrichment score increases when it encounter genes that belong to the gene-set, and decreases otherwise. The enrichment score represents the maximum deviation from 0 during the random walk (Subramanian et al., 2005).

Metabolomic flux analysis

To measure glycolytic flux, cells were resuspended in 50µl Seahorse XF Base Medium (Agilent) supplemented with 2mM L-glutamine (Thermo Fisher) and incubated for 30min at 37°C in a CO_2–free incubator. Subsequently, 130 µL medium were added and cells were incubated for 1h. ECAR was measured using the Seahorse XFe96 metabolic flux analyzer (Agilent). Cells were sequentially treated with 10mM glucose (Sigma), 1µM oligomycin (Agilent), and 30mM 2-deoxy-D glucose (2DG) (Sigma). Oligomycin is an inhibitor of mitochondrial ATPase and 2DG inhibits the glycolytic enzyme hexokinase II. To assess mitochondrial function, cells were resuspended in 50µL Seahorse XF Base Medium (Agilent) supplemented with 2mM L-glutamine (Thermo Fisher), 1mM sodium pyruvate (Thermo Fisher), 10mM glucose (Sigma) and incubated for 30min at 37°C in a CO_2–free

incubator. Subsequently, 130µL medium were added and cells were incubated for an additional 1h. Cells were sequentially treated with 1µM oligomycin (Agilent), 1µM FCCP (Agilent) and 1µM rotenone+ antimycin (Agilent). OCR was measured using the Seahorse XFe96 metabolic flux analyzer (Agilent).

Further Materials are listed in Dataset S4

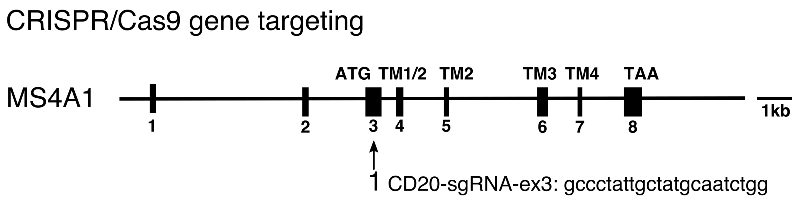

Fig. S1

CRISPR/Cas9 CD20KO generation. Schematic of CRISPR-mediated gene targeting site in exon 3 of the scaled exon-intron organization of the human *MS4A1* gene. Sequence of CRISPR- single guide RNA (sgRNA) as depicted.

The BCR and CD19 are indispensable for CD20KO B cell activation

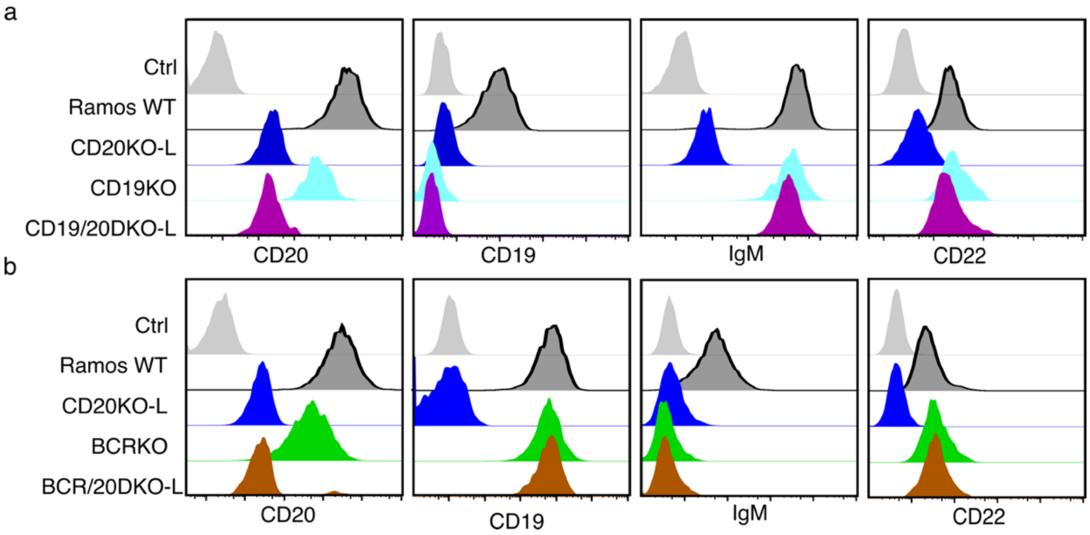

Fig. S2.

Flow cytometry analysis showing surface molecule expression on a CD19/20DKO-L, CD19KO, and CD20KO-L Ramos cells compared to WT and unstained control (grey) or b on BCR/20DKO-L, BCRKO, CD20KO-L Ramos cells compared to WT and unstained control (in grey).

Loss of CD20 leads to B cell activation via the canonical PI3K pathway

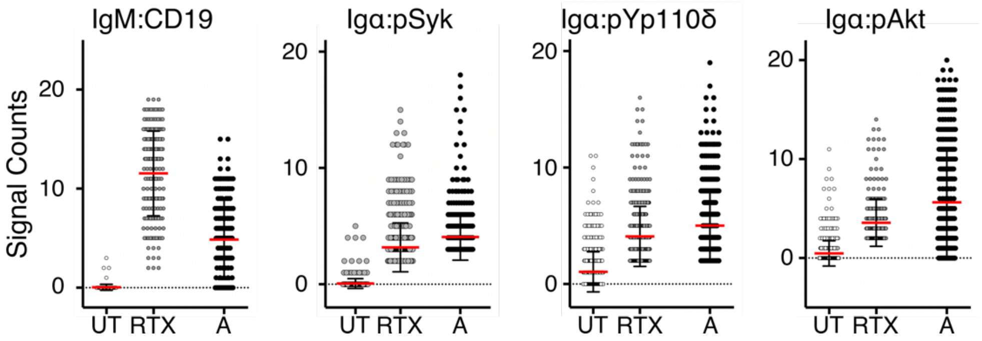

Fig S3

RTX treatment activates primary naïve B cells via the canonical BCR pathway

PLA on resting (UT), 15 min RTX treated (RTX) or 5 min pervanadate activated (A) negatively selected naïve B cells from the peripheral blood of a healthy donor. Scatter dot plots showing the mean and SD of quantified results of the proximity of CD19 to IgM-BCR, Igα to phosphoSyk-Y525,526 (pSyk), Igα to phospho-p110δ-Y524 (pYp110δ), and of Igα to phosphoAkt-Ser473 (pAkt), n=4.

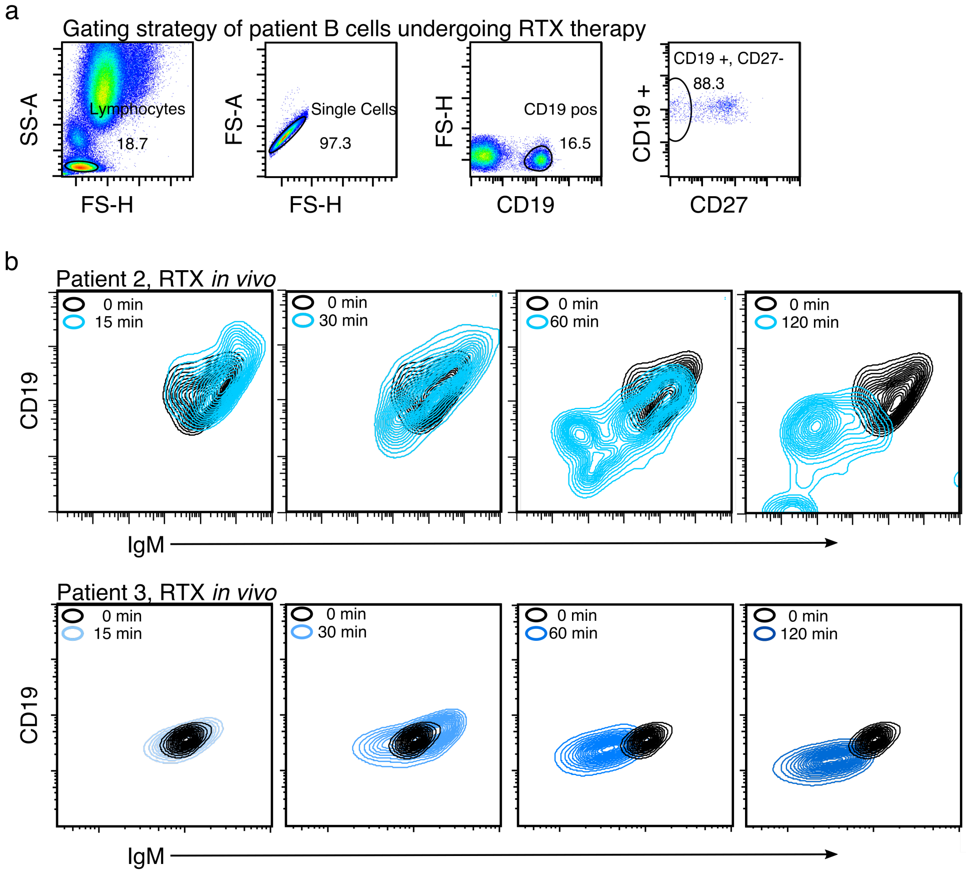

Fig S4.

a Gating strategy for B cell flow cytometry. CD19+ B cells were negatively selected for differential expression CD38 (Plasmablasts) and CD27 (switched memory B cells and plasma cells). **b** Rituximab therapeutic application of RA patient 2 and patient 3 and measurement of B cell surface receptor expression. B cells taken from EDTA whole blood samples of RTX treatment after 0, 15, 30, 60, 120 min. RTX [1mg/mL] flow rate 50mL/h. Flow cytometry staining of CD19+/ CD27-/ IgM+ selected B cells show internalization of IgM and CD19.

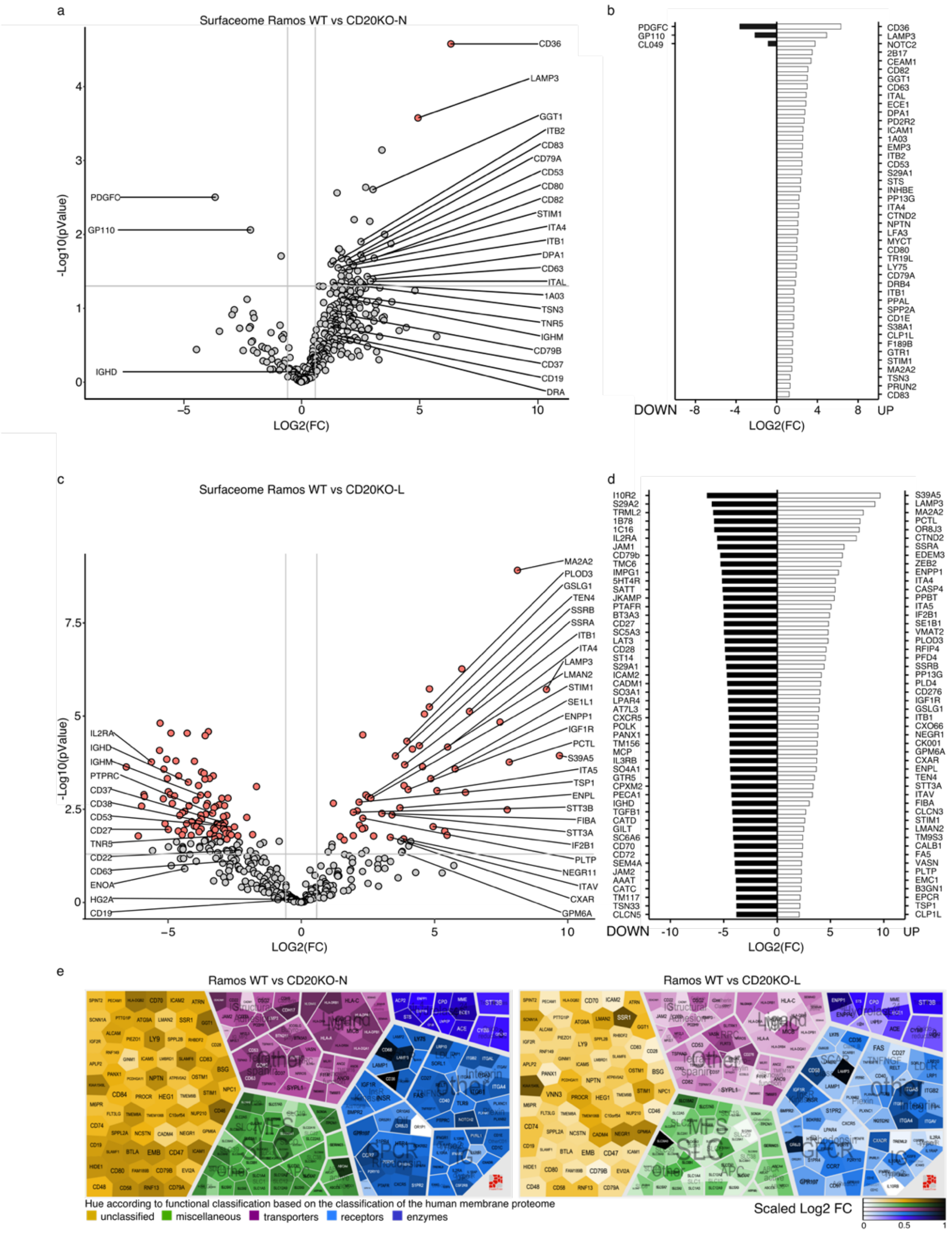

Fig S5

Surfaceome analysis of CD20KO-N and CD20KO-L Ramos cells. **a** Volcano plot showing the Log2 FC of KO-N cell surface proteins compared to Ramos WT cells 10 days after gene targeting. **b** Top50 of significant (p-value< 0.05) of up (right) or downregulated (left) KO-N cell surface proteins compared to WT Ramos cells. **c** Volcano plot showing the Log2 FC of KO-L cell surface proteins compared to Ramos WT cells 1 month after gene

targeting. **d** Top50 of significant (p-value< 0.05) of up (right) or downregulated (left) KO-L cell surface proteins compared to WT Ramos cells. **e** Voronoi tree map of KO-N surfaceome (left) or KO-L (right). All quantified proteins were mapped and hierarchically grouped according to their functional classification. For representation the Log2 FC values for each protein have been scaled from 0-1, represented by color intensity. The colors represent the Log2 FC between the compared conditions.

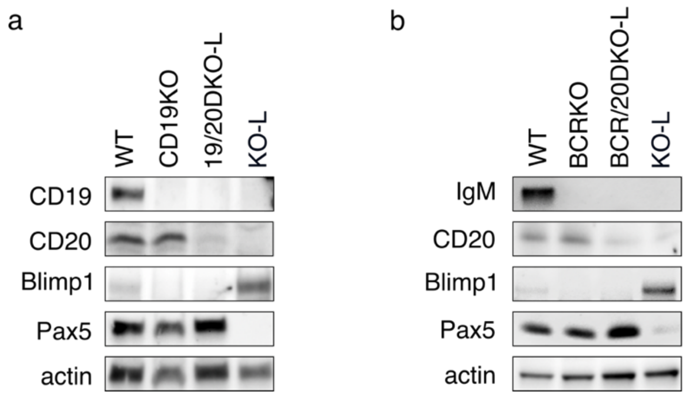

Fig S6

Expression of Pax5 and Blimp1 remain unchanged in CD19/20DKO and BCR/20DKO Ramos cells. **a** Representative examples of western blot analysis for B cell differentiation markers Pax5 and Blimp1 of CD19KO, CD19/20DKO-L, and KO-L Ramos B cells compared to WT. **b** Western blot analysis showing B cell differentiation markers Pax5 and Blimp1 of BCRKO, BCR/20DKO-L, and KO-L Ramos B cells compared to WT. All lysates were taken 20 days after induction of CD20 KO.

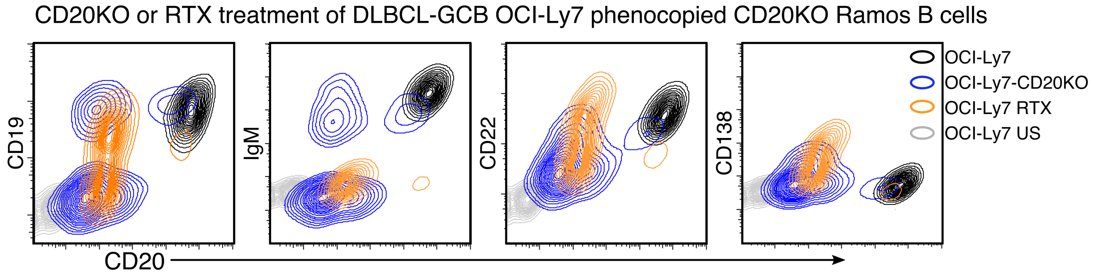

Fig S7.

Flow cytometry analysis of expression of CD20, CD19, CD22, IgM-BCR and CD138 on the surface of either untreated DLBCL-GCB OCI-Ly7 B cell type (black) or upon constant RTX treatment for 6 days (10µg/mL, orange) or after day 8 of CRISPR/Cas9 induced CD20KO (blue). Transfected OCI-Ly7 B cells were left unsorted to visualize the different kinetics of surface receptor abundance upon loss of CD20. The unstained transfection control is shown in grey.

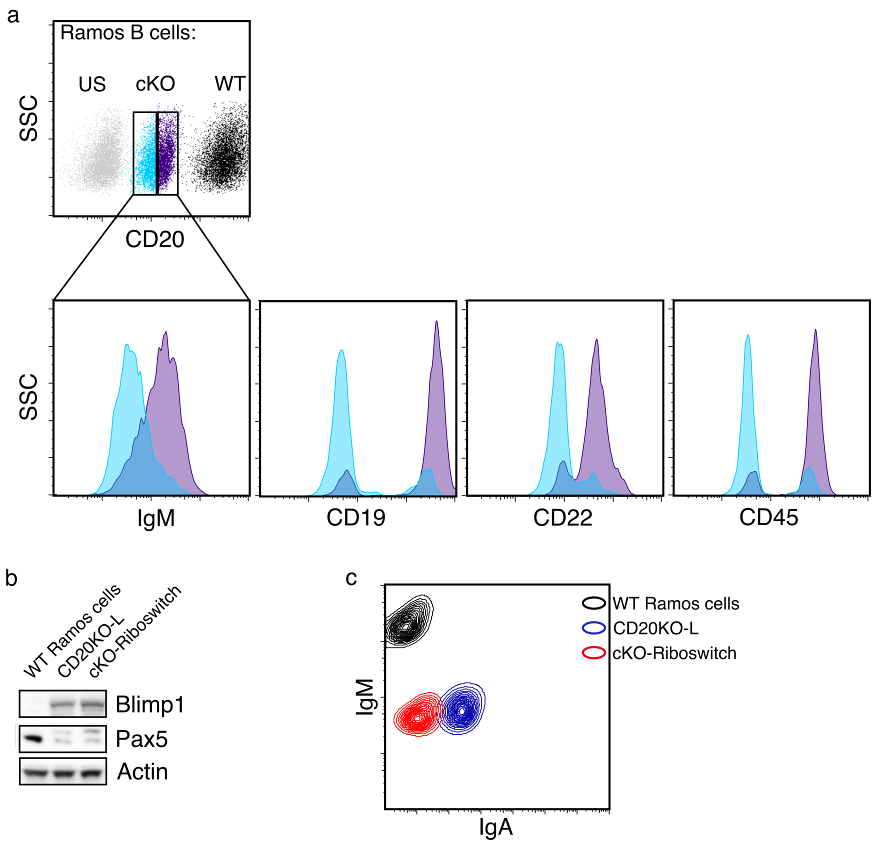

Fig S8

Expression of surface receptors on cKO Ramos cells is dependent on residual amounts of CD20. **a** Flow cytometry analysis of expression of CD20, IgM-BCR, CD19, CD22, and CD138 on the surface of either WT (black) or cKO Ramos cells 2 weeks after transfection with the c-exon. CD20 surface expression of cKO Ramos cells is divided in gated according to the expression level (light-blue vs purple), unstained control in grey. **b** Extended Fig 5d. Western blot of lysates of cKO and CD20KO-L Ramos cells, taken 20 days after transfection of CD20KO, showing PAX5 to BLIMP-1 transcriptional switch compared to WT Ramos cells. **c** flow cytometry analysis 3 weeks after transfection showing the IgA-expression of cKO (red) compared to CD20KO-L (blue) and WT Ramos B cells (black).

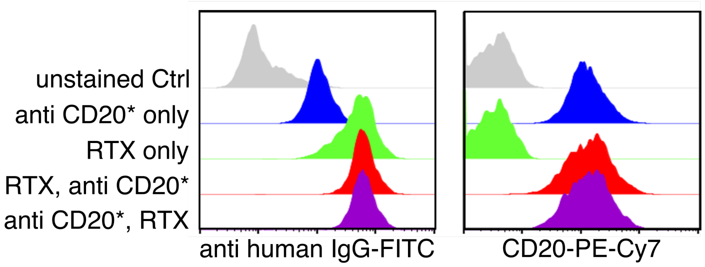

Fig. S9.

CD20 staining control. Flow cytometry control for equal staining of CD20 with rituximab and anti-human CD20-PECy7. Treatment with RTX [10µg/mL] for 1 min on ice, co-staining with anti CD20-PECy7 for 5 min on ice, or vice versa. Washing with ice-cold PBS.

Dataset S1 (separate file). Selected plasma cell specific genes and expression

Selected plasma cell specific genes and expression in KO-I, KO-L, and WT Ramos cells as well as annotated references, used in the transcriptome comparison of differentiated CD20KO Ramos cell lines versus WT Ramos cells and CLL B cell before versus after RTX treatment.
FC- fold change, adj- adjusted, KO-I- KO intermediate, KO-L- KO late.

Ref.-References:
a https://www.ncbi.nlm.nih.gov/gene;
b http://www.genomicscape.com/microarray/nbtopc.php;
c doi: 10.4049/jimmunol.1103720;
d doi:10.18632/oncotarget.8982;
e http://amp.pharm.mssm.edu/Harmonizome/gene_set/plasma+cell/TISSUES+Curated+Tissue+Protein+Expression+Evidence+Scores;
f doi:10.1111/ejh.12779

Dataset S2 (separate file). PLASMA_UP_fgsea_summary

Statistical significance of the enrichment score of every individual patient from GSE37168. ES: enrichment score, NES: normalized enrichment score

Dataset S3 (separate file). Analysis of Metabolites

Metabolomic analysis of CD20KO Ramos cells cultivated in glucose or galactose compared to Ramos WT cells. Analysis by Metabolon-05-18VW.

Dataset S4 (separate file). Further Material as not referenced before.

List of used antibodies, primers and oligonucleotides, chemicals, biological samples, used software and algorithms, provided online datasets.

References.

Almén, M.S., Nordström, K.J.V., Fredriksson, R., and Schiöth, H.B. (2009). Mapping the human membrane proteome: A majority of the human membrane proteins can be classified according to function and evolutionary origin. BMC Biol. *7*, 50.
Subramanian, A., Tamayo, P., Mootha, V.K., Mukherjee, S., Ebert, B.L., Gillette, M.A., Paulovich, A., Pomeroy, S.L., Golub, T.R., Lander, E.S., et al. (2005). Gene set enrichment analysis: A knowledge-based approach for interpreting genome-wide expression profiles. Proc. Natl. Acad. Sci. *102*, 15545 LP – 15550.
